# Poor ICU performance is most often due to increasingly ageing population (>60 years) - is this true? an analysis in Indian scenario

**DOI:** 10.1186/2197-425X-3-S1-A533

**Published:** 2015-10-01

**Authors:** A Kar, A Datta, A Ahmed

**Affiliations:** Medica Superspecialty Hospital, Medica Institute of Critical Care, Kolkata, India

## Introduction

Increasing numbers of patients over 60 years are being admitted to the intensive care unit (ICU). After adjustment for disease severity, ICU mortality rates are higher in elderly patients than in younger populations which can impact an ICU performance.

## Objectives

The aim of our study was to analyze whether poor ICU Performance based on SMR (Standardized Mortality Ratio) in an ICU is related to increasingly ageing population (>60 years). The study was conducted in a level 3 care ICU of a tertiary Hospital in Kolkata.

## Methodology

2374 patients (>20 years) admitted in a level 3 care ICU over a period of one year were included in the study. Readmission cases were not counted. Entire group(Group A) was divided into 3 subgroups based on age- Group B (21-40 years), Group C (41-60 years) and Group D (>60 years). Outcome assessment was done in the entire group and subgroups using APACHE 4 Predictive Mortality Model. Demographic data, number of observed deaths, Predicted Mortality Rate (PMR), Standardized Mortality Ratio (SMR) was documented in the entire group and all subgroups. Comparisons between ageing population (Group D) with rest were done to analyze whether ICU performance based on SMR is primarily affected due to ageing population.

## Results

Entire group (Group A) (2374 cases), Mean age was 60.41 years ± 13.17SD (Median 62), Mean A4 was 47.72 ± 31.53SD (Median 39), PMR was 13.63, Observed deaths 325 and SMR 1.004 (CI 0.90-1.12). Among Subgroups- Group B (221 cases), Mean age was 32.52 years ± 5.81SD (Median 34), Mean A4 was 40.68 ± 32.68SD (Median 32), PMR was 10.69, Observed deaths 30 and SMR 1.27 (CI 0.87-1.79). Group C (839 cases), Mean age was 52.88 years ± 5.41SD (Median 54), Mean A4 was 41.39 ± 30.82SD (Median 30), PMR was 11.26, Observed deaths 100 and SMR 1.06 (CI 0.86-1.28). Group D (1314 cases), Mean age was 69.91 years ± 5.61SD (Median 70), Mean A4 was 52.95 ± 30.83SD (Median 44), PMR was 15.64, Observed deaths 195 and SMR 0.95 (CI 0.82-1.09). On analysis APACHE 4 Score was significantly higher in Group D (>60 years) when compared to Entire group (Group A p≤0.0001), Group B (21-40 years) (p≤.0001) and Group C (41-60 years) (p≤.0001). SMR in the ageing group (Group D) was lower (0.95) as comparison to Group A (1.004)(Entire Group), subgroup Group B (1.27) and subgroup Group C (1.06).

## Conclusions

SMR was significantly lower in ageing group (Group D) in our analysis and age itself explains only a small part of the increased hospital mortality. Quality of care and proper distribution of available resources are probably more important while relating outcome.
